# Supplementary figures and images for: Expression and immunological role of FUNDC2 in pan-cancer
Source: PLoS One. 2025 Apr 28;20(4):e0319343. doi: 10.1371/journal.pone.0319343 (PMC12036908; doi:10.1371/journal.pone.0319343)

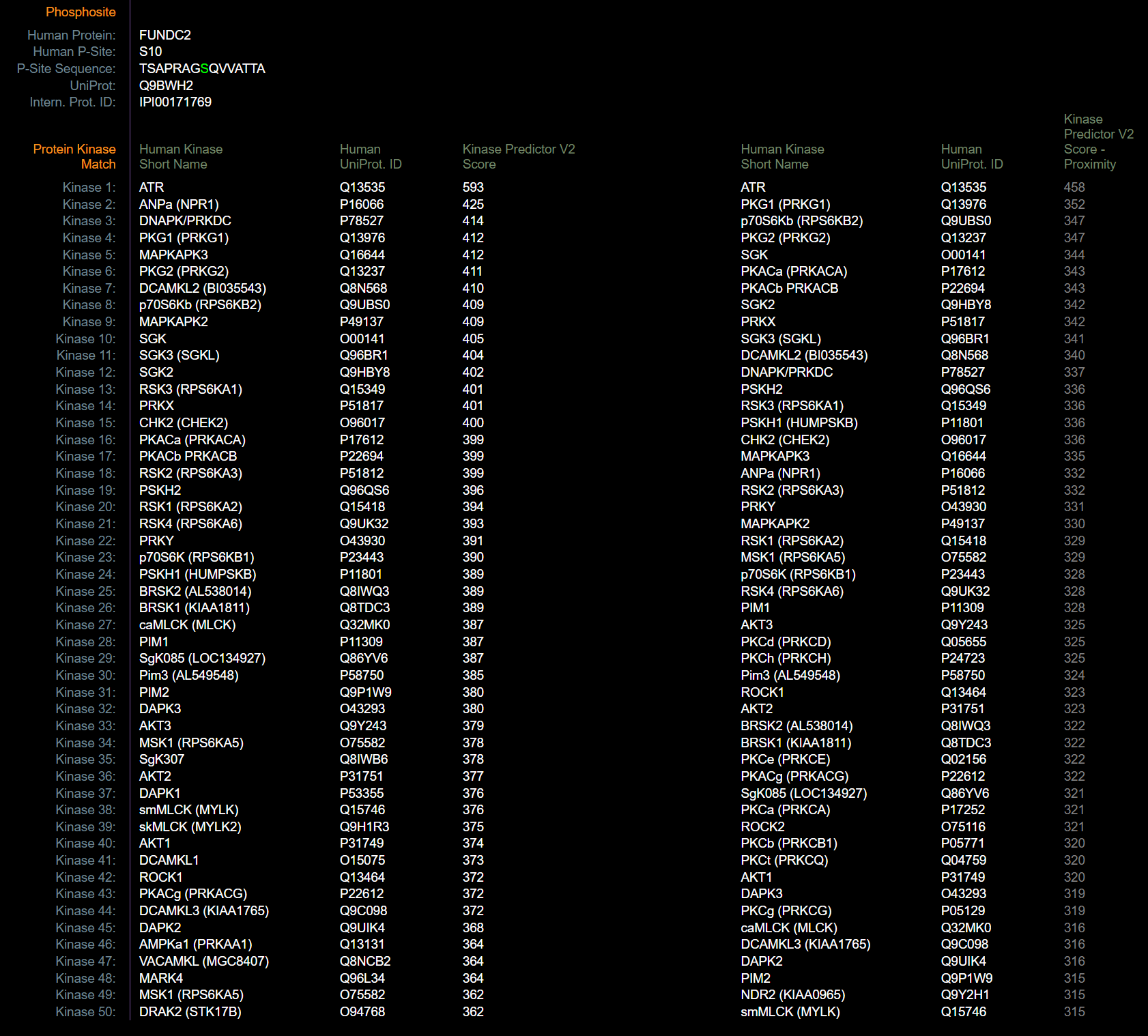

Supplement: S1 Fig — The PhosphoNet (http://www.phosphonet.ca/) is employed to predict the possible protein kinases responsible for FUNDC2 at the specific site of Ser10 (TSAPRAGSQVVATTA). The data show that ataxia telangiectasia and Rad3-related protein (ATR, ATR serine/threonine kinase) is most likely the protein kinase for FUNDC2 at Ser10. (TIF) [file pone.0319343.s001.tif]
